# Supplementary material for: Tracing G-Protein-Mediated Contraction and Relaxation in Vascular Smooth Muscle Cell Spheroids
Source: Cells. 2022 Dec 28;12(1):128. doi: 10.3390/cells12010128 (PMC9818396; doi:10.3390/cells12010128)
Supplement: Supplementary file 1 [file cells-12-00128-s001.zip › Supplement - Garg et al-revised.pdf]

Figure S1

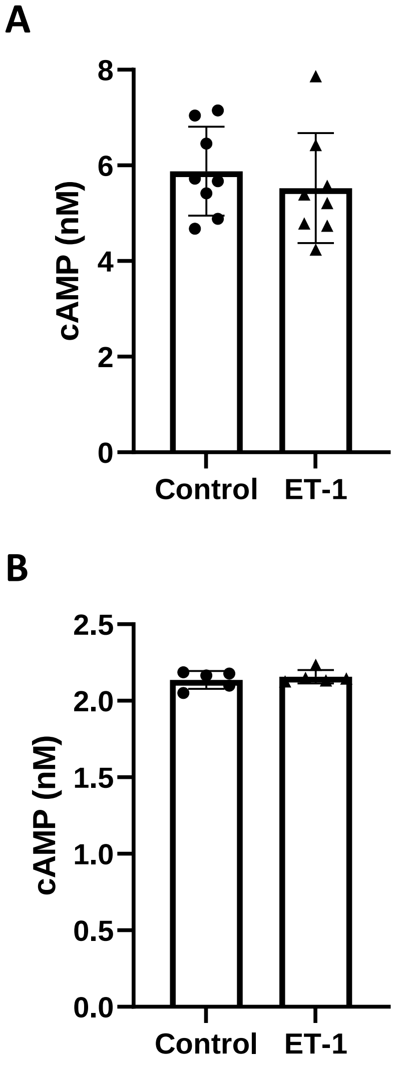

Analysis of cAMP levels in murine aoSMCs cultured as monolayer (2D) or as spheroids (3D)
The cAMP content was measured in 2D (A, n=8) or 3D aoSMC (B, n=5) treated with either solvent (control) or ET-1 (100 nM).

Figure S2

Gene	Name	log2 FC (3D vs. 2D)	adj. p-value	log2 FC (3D +TGFβ1 inh. vs. 3D)	adj. p-value
ADRA1A	adrenoceptor alpha 1A	0.09	>0.05	-0.12	>0.05
ADRA1B	adrenoceptor alpha 1B	0.09	>0.05	-0.08	>0.05
ADRA1D	adrenoceptor alpha 1D	-0.02	>0.05	0.23	>0.05
ADRA2A	adrenoceptor alpha 2A	-0.23	>0.05	0.05	>0.05
ADRA2B	adrenoceptor alpha 2B	0.08	>0.05	0.08	>0.05
ADRA2C	adrenoceptor alpha 2C	0.34	>0.05	0.03	>0.05
ADRB1	adrenoceptor beta 1	-0.06	>0.05	-0.18	>0.05
ADRB2	adrenoceptor beta 2	-0.13	>0.05	0.02	>0.05
ADRB3	adrenoceptor beta 3	0.03	>0.05	-0.12	>0.05
EDNRA	**endothelin receptor type A**	**2.45**	**0.012**	-0.56	**0.017**
EDNRB	endothelin receptor type B	0.12	>0.05	0.06	>0.05
GNA11	G protein subunit alpha 11	-0.43	0.046	0.10	>0.05
GNA12	G protein subunit alpha 12	0.21	>0.05	-0.06	>0.05
GNA13	G protein subunit alpha 13	0.68	0.048	-0.65	0.008
GNAI1	G protein subunit alpha i1	0.33	>0.05	-0.43	>0.05
GNAI2	G protein subunit alpha i2	-0.48	0.042	-0.01	>0.05
GNAI3	G protein subunit alpha i3	-0.45	0.049	-0.02	>0.05
GNAQ	G protein subunit alpha q	-0.21	>0.05	0.10	>0.05
GNAS	GNAS complex locus	0.49	0.016	-0.22	>0.05

RNA expression analyses of human VSMCs

(A) RNA was isolated from human umbilical artery smooth muscle cells (HUASMCs) cultured as 2D monolayer or 3D spheroids or 3D spheroids treated with the TGFRI/RII inhibitor LY2109761 were subjected to transcriptome analysis applying a whole genome microarray (n=3). The table summarizes the expression regulation of selected genes. (green: down-regulation, red: up-regulation, 3D vs 2D (left), 3D control vs 3D +LY2109761 (right).

Figure S3

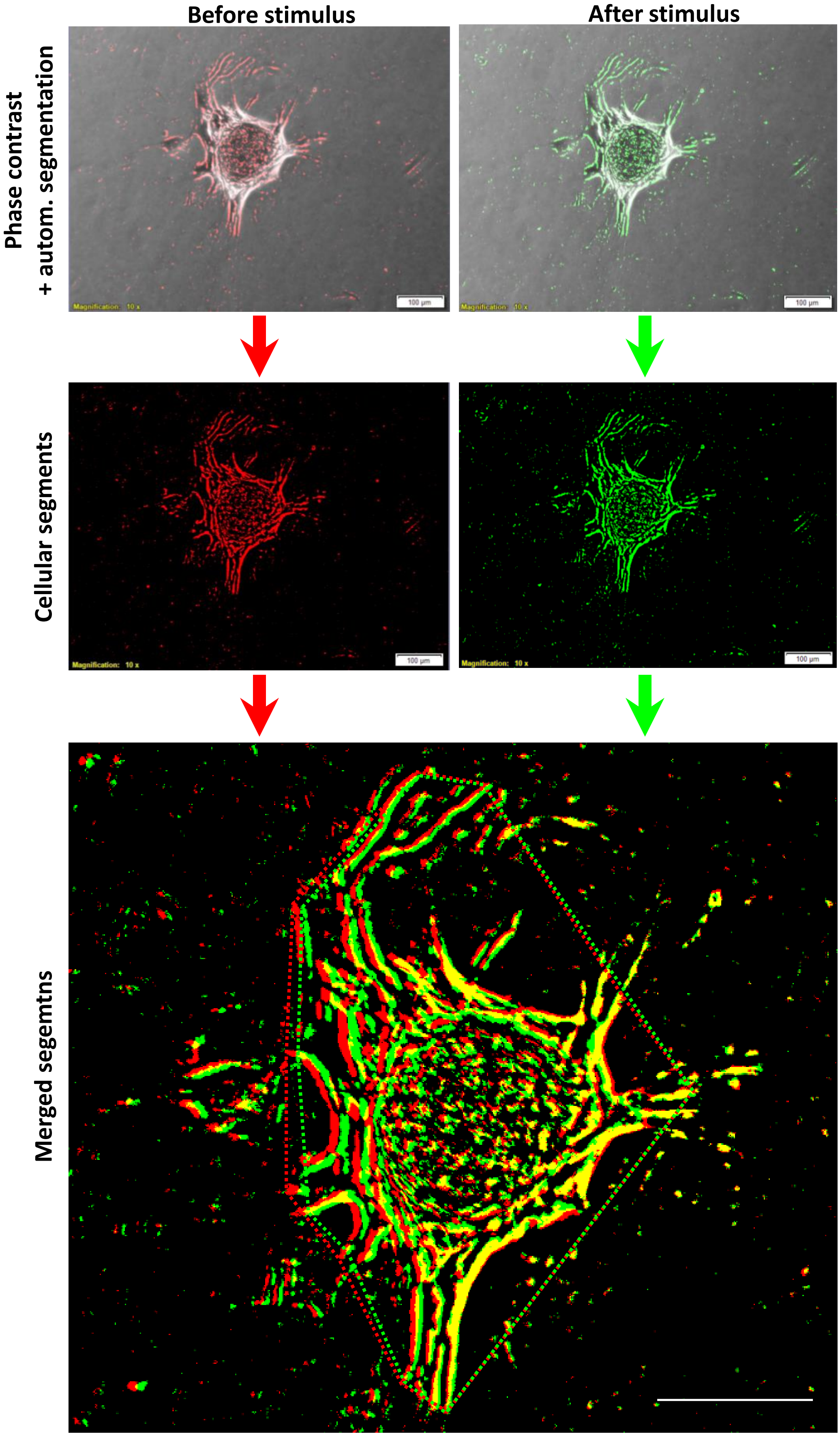

Assessment of the contraction of a VSMC spheroid attached to a collagen gel

Exemplary results showing a murine aoSMC spheroid attached to a collagen type I hydrogel before (red) and after (green) exposure to KCl. Cellular segments were detected by computer-aided morphometry of phase-contrast images. Cellular segments were isolated and merged. Area changes induced by contraction/relaxation were determined by overlaying both images and connecting segmental fix points defining the outer perimeter of attached VSMCs before and after the stimulus (scale bars: 100 µm).

Figure S4

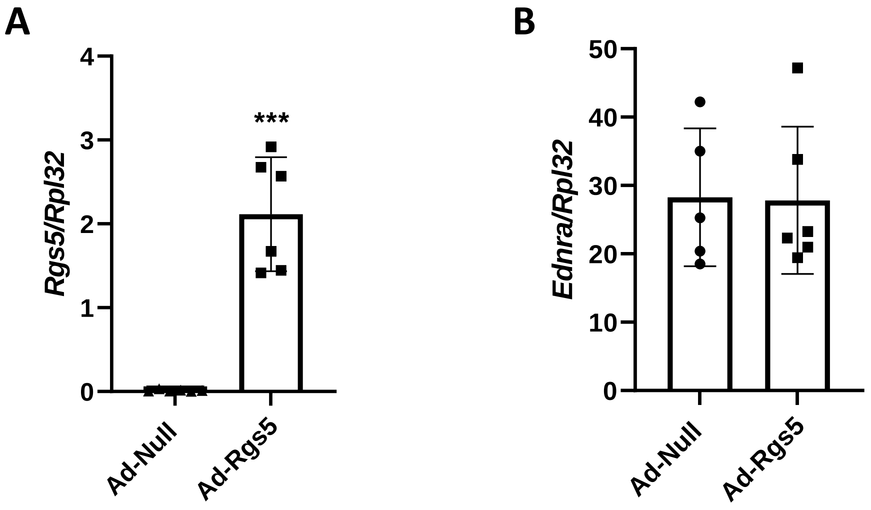

Overexpression of *Rgs5* in murine VSMCs

Murine aoSMC were transduced with adenoviral vectors carrying either empty vector (Ad-Null) or Rgs5 (Ad-Rgs5). 3D spheroids were subsequently generated and the mRNA content of either *Rgs5* (A, n=6, ***p<0.001) or *Ednra* (B, n=5-6) was quantified by qPCR. *RPL32* was used as a reference.

Figure S5

Each antibody is spotted in duplicate vertically

	A	B	C	D	E	F	G	H
1<br>2	**POS**	**POS**	**NEG**	**NEG**	**Akt (P-S473)**	**CREB (P-S133)**	**ERK1 (P-T202/Y204) ERK2 (P-Y185/Y187)**	**GSK3a (P-S21)**
3<br>4	**GSK3b (P-S9)**	**HSP27 (P-S82)**	**JNK (P-T183)**	**MEK (P-S217/221)**	**MKK3 (P-S189)**	**MKK6 (P-S207)**	**MSK2 (P-S360)**	**mTOR (P-S2448)**
5<br>6	**p38 (P-T180/Y182)**	**P53 (P-S15)**	**P70S6K (P-T421/S424)**	**RSK1 (P-S380)**	**RSK2 (P-S386)**	**NEG**	**NEG**	**POS**

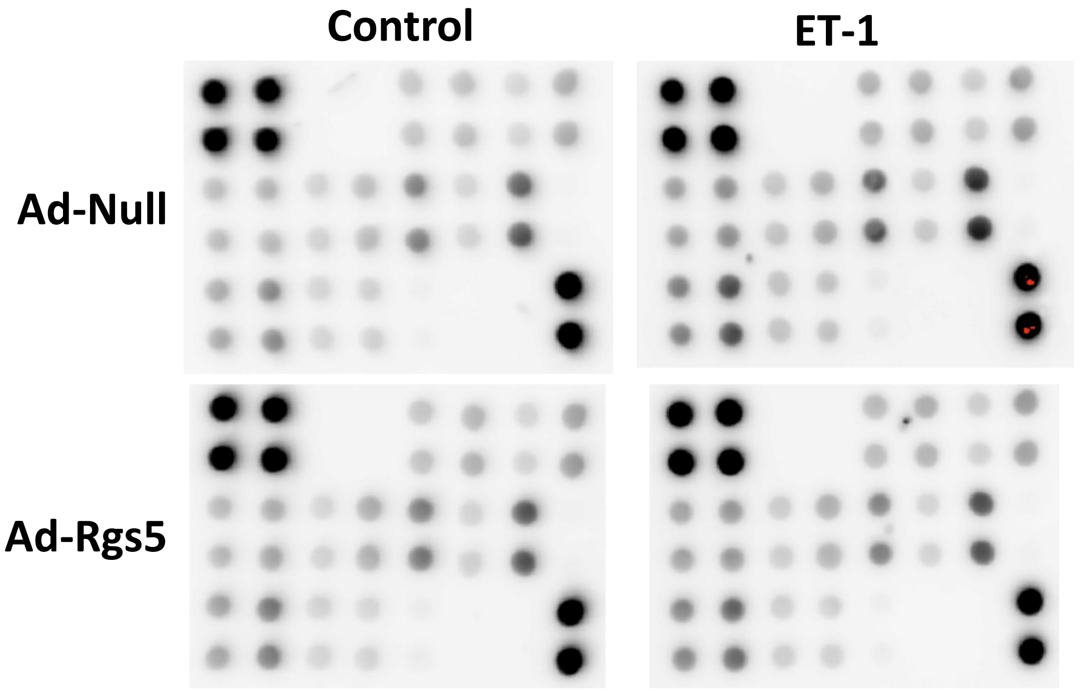

Representative array (Figure 4)

The phosphorylation level of multiple kinases was analyzed by an array (AAH-MAPK, RayBiotech) outlined as indicated in the table (phosphorylation sites in brackets). Each target is represented by two spots. The mean grey intensity of these spots was quantified using the Image Quant TL Software (GE Healthcare, Version 8.1). Representative arrays from control (Ad-Null) and *Rgs5* overexpressing (Ad-Rgs5) VSMCs treated with solvent (control) or ET-1 are shown below the table.
